# Neurocognitive Analysis of Low-level Arsenic Exposure and Executive Function Mediated by Brain Anomalies Among Children, Adolescents, and Young Adults in India

**DOI:** 10.1001/jamanetworkopen.2023.12810

**Published:** 2023-05-12

**Authors:** Nilakshi Vaidya, Bharath Holla, Jon Heron, Eesha Sharma, Yuning Zhang, Gwen Fernandes, Udita Iyengar, Alex Spiers, Anupa Yadav, Surajit Das, Sanjit Roy, Chirag K. Ahuja, Gareth J. Barker, Debasish Basu, Rose Dawn Bharath, Matthew Hickman, Sanjeev Jain, Kartik Kalyanram, Kamakshi Kartik, Murali Krishna, Ghattu Krishnaveni, Kalyanaraman Kumaran, Rebecca Kuriyan, Pratima Murthy, Dimitri Papadopoulos Orfanos, Meera Purushottam, Sunita Simon Kurpad, Lenin Singh, Roshan Singh, B. N. Subodh, Mireille Toledano, Henrik Walter, Sylvane Desrivières, Amit Chakrabarti, Vivek Benegal, Gunter Schumann

**Affiliations:** 1 Centre for Population Neuroscience and Precision Medicine, Department of Psychiatry and Neuroscience, Charité Universitätsmedizin Berlin, Berlin, Germany; 2 Centre for Addiction Medicine, National Institute of Mental Health and Neurosciences, Bangalore, India; 3 Department of Psychiatry and Integrative Medicine, National Institute of Mental Health and Neurosciences, Bangalore, India; 4 Centre for Public Health, Bristol Medical School, University of Bristol, Bristol, United Kingdom; 5 Department of Child & Adolescent Psychiatry, National Institute of Mental Health and Neurosciences, Bangalore, India; 6 Social, Genetic and Developmental Psychiatry Centre, Institute of Psychiatry, Psychology & Neuroscience, King’s College London, London, United Kingdom; 7 Population Health Sciences, Bristol Medical School, University of Bristol, Bristol, United Kingdom; 8 Institute of Psychology, Psychiatry & Neuroscience, King’s College London, London, United Kingdom; 9 MRC (Medical Research Council) Centre for Environment and Health, Imperial College London, London, United Kingdom; 10 Indian Council of Medical Research–Centre on Noncommunicable Diseases, Kolkata, India; 11 Department of Radiodiagnosis and Imaging, Postgraduate Institute of Medical Education and Research, Chandigarh, India; 12 Department of Neuroimaging, Institute of Psychology, Psychiatry & Neuroscience, King’s College London, London, United Kingdom; 13 Department of Psychiatry, Postgraduate Institute of Medical Education and Research, Chandigarh, India; 14 Department of Neuroimaging and Interventional Radiology, National Institute of Mental Health and Neurosciences, Bangalore, India; 15 Department of Psychiatry, National Institute of Mental Health and Neurosciences, Bangalore, India; 16 Rishi Valley, Rural Health Centre, India; 17 Foundation for Research and Advocacy in Mental Health, Mysore, India; 18 Epidemiology Research Unit, CSI Holdsworth Memorial Hospital, Mysore, India; 19 Division of Nutrition, St John’s Research Institute, Bangalore, India; 20 NeuroSpin, Commissariat à l’Énergie Atomique et aux Énergies Alternatives, Université Paris-Saclay, Paris, France; 21 Molecular Genetics Laboratory, National Institute of Mental Health and Neurosciences, Bangalore, India; 22 Department of Psychiatry, St John’s Medical College and Hospital, Bangalore, India; 23 Department of Medical Ethics, St John’s Medical College and Hospital, Bangalore, India; 24 Regional Institute of Medical Sciences, Imphal, Manipur, India; 25 Mohn Centre for Children’s Health and Wellbeing, School of Public Health, Imperial College London, London, United Kingdom; 26 Psychiatry, Psychiatric Neuroscience and Neurophilosophy, Research Division of Mind and Brain, Charité-Universitätsmedizin Berlin, Berlin, Germany; 27 Centre for Population Neuroscience and Precision Medicine, Institute for Science and Technology of Brain-Inspired Intelligence, Fudan University, Shanghai, China

## Abstract

**Importance:**

Arsenic, a contaminant of groundwater and irrigated crops, is a global public health hazard. Exposure to low levels of arsenic through food extends well beyond the areas with high arsenic content in water.

**Objective:**

To identify cognitive impairments following commonly prevalent low-level arsenic exposure and characterize their underlying brain mechanisms.

**Design, Setting, and Participants:**

This multicenter population-based cohort study analyzed cross-sectional data of the Indian Consortium on Vulnerability to Externalizing Disorders and Addictions (cVEDA) cohort, recruited between November 4, 2016, and May 4, 2019. Participants aged 6 to 23 years were characterized using deep phenotyping measures of behavior, neuropsychology, psychopathology, brain neuroimaging, and exposure to developmental adversities and environmental neurotoxins. All analyses were performed between June 1, 2020, and December 31, 2021.

**Exposure:**

Arsenic levels were measured in urine as an index of exposure.

**Main Outcomes and Measures:**

Executive function measured using the cVEDA neuropsychological battery, gray matter volume (GMV) from T1-weighted magnetic resonance imaging, and functional network connectivity measures from resting state functional magnetic resonance imaging.

**Results:**

A total of 1014 participants aged 6 to 23 years (589 male [58.1%]; mean [SD] age, 14.86 [4.79] years) were included from 5 geographic locations. Sparse-partial least squares analysis was used to describe a negative association of arsenic exposure with executive function (*r* = −0.12 [*P* = 5.4 × 10^−4^]), brain structure (*r* = −0.20 [*P* = 1.8 × 10^−8^]), and functional connectivity (within network, *r* = −0.12 [*P* = 7.5 × 10^−4^]; between network, *r* = −0.23 [*P* = 1.8 × 10^−10^]). Alterations in executive function were partially mediated by GMV (*b* = −0.004 [95% CI, −0.007 to −0.002]) and within-network functional connectivity (*b* = −0.004 [95% CI, −0.008 to −0.002]). Socioeconomic status and body mass index moderated the association between arsenic and GMV, such that the association was strongest in participants with lower socioeconomic status and body mass index.

**Conclusions and Relevance:**

The findings of this cross-sectional study suggest that low-level arsenic exposure was associated with alterations in executive functioning and underlying brain correlates. These results indicate potential detrimental consequences of arsenic exposure that are below the currently recommended guidelines and may extend beyond endemic risk areas. Precision medicine approaches to study global mental health vulnerabilities highlight widespread but potentially modifiable risk factors and a mechanistic understanding of the impact of low-level arsenic exposure on brain development.

## Key Points

**Question:**

What is the association of low-level arsenic exposure with brain structure and function and subsequent cognitive functioning?

**Findings:**

This cohort study of 1014 participants describes a syndrome of impairments in executive function associated with low-level arsenic exposure and characterizes its underlying brain mechanisms. The arsenic-associated impairments are exacerbated by risk factors such as poor nutrition and poverty.

**Meaning:**

These findings call for a reexamination of the definitions of safe levels of environmental arsenic exposure; improvements in nutrition and socioeconomic conditions may additionally serve as potential mechanisms to ameliorate the harmful consequences of environmental arsenic insults.

## Teaser

This cohort study investigates potential arsenic-related syndromic alterations across multiple cognitive domains and associations of arsenic exposure with brain structure and function among children, adolescents, and young adults in India.

## Introduction

Arsenic is a highly toxic natural component of the earth’s crust, and arsenic exposure is a major environmental public health concern worldwide.^1,2^ In Asia, arsenic-laden rocks in the Himalayas feed 4 major river systems: the Ganges-Brahmaputra, Irrawaddy, Mekong, and Red.^3^ The catchment areas of these river systems are the world’s major rice-growing regions.^4^ Aquifers contaminated with inorganic arsenic provide drinking water and are also widely used for cultivation. This results in increased arsenic levels in staples,^5^ with a 10-fold increase reported in rice.^6,7^ Rice and other staples are then distributed throughout the region and across the world, extending arsenic exposure far beyond the river catchment areas. Depending on food habits, this distribution can result in marked increases in arsenic exposure. While the average US concentration of arsenic in urine is 5.96 µg/L (to convert to µmol/L, multiply by 0.0133), this number increases to 14.3 μg/L in Asian American individuals,^8^ for whom rice is often the main dietary staple.

Most studies investigating chronic arsenic toxic effects have examined exposure to high doses.^9,10^ A study of adolescents in Bangladesh measured a mean urinary arsenic concentration of 205.3 μg/L, with individual concentrations as high as 1312 μg/L.^11^ In contrast, mean (geometric) urinary arsenic levels in the US are 4.89 μg/L for children aged 6 to 11 years and 5.00 μg/L for those aged 12 to 19 years.^8^ Similar levels were measured in children and adolescents from Canada^12^ and Germany,^13^ whereas studies reported a mean urine concentration of 9.57 μg/L in China.^14^

Apart from skin lesions and increased risk for certain cancers and peripheral neuropathies, reports also describe cognitive impairments following chronic exposure.^2,9^ Arsenic exposure in children and adolescents has been associated with impaired attention,^15,16^ working memory,^15,16,17^ verbal comprehension,^17^ and reasoning.^15,17^ These impairments are likely to contribute to a decrease in IQ^11,17^ and are suggestive of a wider cognitive syndrome. We applied multivariate statistical modeling to (1) investigate potential association of arsenic exposure with syndromic alterations across multiple cognitive domains; (2) identify association of arsenic exposure with brain structure and function; (3) understand the association between arsenic and brain and cognitive alterations, and (4) explore the moderating influence of other measures of environmental risk and physical health.

## Methods

### Study Population

This study followed the Strengthening the Reporting of Observational Studies in Epidemiology (STROBE) reporting guideline. The Indian Consortium on Vulnerability to Externalizing Disorders and Addictions (cVEDA) is an accelerated longitudinal cohort of 9010 participants aged 6 to 23 years recruited from 7 sites across India.^18,19^ The cVEDA study was approved by the institutional ethics review boards of all collaborating institutions. Written informed consent was obtained from participants 18 years and older and from parents of participants younger than 18 years (along with assent from minors). Herein we analyze cross-sectional data of a subgroup of 1014 participants recruited between November 4, 2016, and May 4, 2019, at 5 sites who had neuroimaging assessments in addition to urinary arsenic measurement. Their dietary intake was measured as the frequency of consumption on different items of the Short Food Questionnaire. Details are provided in eMethods 1 in Supplement 1.

### Cognition

Cognitive skills were measured using the cVEDA neuropsychological battery (eMethods 1 in Supplement 1). Briefly, we applied the following tests to measure specific cognitive domains: (1) Balloon Analogue Risk-Taking Task for risk-taking behavior; (2) Corsi Block Tapping Test for visual-spatial attention and working memory; (3) Digit Span Test for attention and working memory; (4) Emotional Recognition Task for emotion recognition; (5) 27-item Now or Later monetary choice questionnaire for reward processing and decision-making; (6) Social Cognition Rating Tools in the Indian Setting for theory of mind and *faux pas*; (7) Stop Signal Task for response inhibition; (8) Trail Making Test for set shifting; and (9) Wisconsin Card Sorting Test for cognitive flexibility.

### Moderators

Body mass index (BMI) was calculated as weight in kilograms divided by height in meters squared. Socioeconomic status (SES) was calculated using the Standard of Living Index, parameter standardized, and validated in the National Family Health Survey of India.^20^ Details are provided in eMethods 1 in Supplement 1.

### Arsenic and Magnetic Resonance Imaging

Urinary arsenic estimation was performed using an atomic absorption spectrometer with a flow injection hydride generator system (AA800; PerkinElmer) and Zeeman-effect background correction. Arsenic levels are expressed as micrograms per liter. Analysis of urinary arsenic levels was performed on the deep phenotyping subgroup who also underwent magnetic resonance imaging (MRI). Regional brain volumes of gray matter derived from T1-weighted MRI and functional network connectivity measures derived from independent component analysis of the resting state functional MRI were investigated. Details are provided in eMethods 1 and eTable 1 in Supplement 1.

### Statistical Analysis

Data analyses were performed between June 1, 2020, and December 31, 2021. We used sparse partial least squares (sPLS) analysis to establish associations among cognition, brain structures, and functions by finding the weighted sum of variables in these data sets that correlated maximally with arsenic. We used 10-fold cross-validation to quantify the strength and significance of these associations. We added sparse formulation to the weighted vectors through the application of L1 penalization, which sets weights of variables with negligible variance to zero. Sparsity was applied under resampling to ensure that the features retained are robustly associated. Significance was ascertained via cross-validation using bootstrapped CIs and permutation testing. Subsequently, we performed mediation analysis. The mediation pathways were defined based on the bootstrapped variability around the product of nonstandardized path coefficient estimates (ie, *b* values). We examined the direct association of arsenic exposure with cognitive performance (path c), the direct association of brain structure and connectivity with cognitive performance (path b), and the indirect association of arsenic exposure with cognitive performance through brain structure and connectivity (path a × b). Next, we conducted moderated mediation analysis using data on the participant’s SES and BMI. For both mediation and moderation, bias-corrected 95% CIs using 10 000 bootstrapped resamples were generated. Statistical significance was determined if CIs did not contain zero. In all analyses, we controlled for age, sex, site, educational level, and total intracranial volume. A detailed description is provided in eMethods 2 in Supplement 1. To provide conceptual overview, post hoc analysis of physical environment and sensitivity analysis on the components of the significant factors in mediation models were conducted, details are given in eMethods 3 and eTables 9 to 11 in Supplement 1. The sPLS models were programmed using the spls software package^21^; moderated mediation pathways were programmed using the lavaan statistical package.^22^

## Results

### The cVEDA Cohort and Arsenic Exposure

Details of study sites are provided in eFigure 1 of Supplement 1. Of the 9010 individuals in the cVEDA study (mean [SD] age, 14.55 [4.61] years; 4707 female [52.2%] and 4303 male [47.8%]), 1014 had complete data on all variables of interest (mean [SD] age, 14.86 [4.79] years; 425 female [41.9%] and 589 male [58.1%]) (eTable 3 in Supplement 1). Demographics of participants with and without cognitive data are provided in eTable 4 of Supplement 1. Urinary arsenic concentration ranged from limit of detection (0.26 μg/L) to 46.50 μg/L, with a mean (SD) of 9.65 (9.40) μg/L (eTable 5 in Supplement 1). These values are comparable with urinary arsenic levels observed in other studies across the globe (eTable 6 in Supplement 1). However, the mean was lower than that seen in participants living in the Ganges-Brahmaputra River catchment areas (eg, Bangladesh). Further, in our sample, the urinary arsenic levels among participants in southern India were higher than those among participants in northern and northeastern India (ie, the Himalayan river catchment areas) (eTable 5 in Supplement 1).

All the study sites within cVEDA are reported as areas with ground water arsenic content less than 10 μg/L,^23^ that is, below the World Health Organization–recommended cutoff for arsenic in drinking water. Elevated urinary arsenic levels could be indicative of dietary intake. Urinary arsenic levels were correlated with higher intake of rice, egg, fish, and chicken (Table 1).

**Table 1.** Association of Arsenic Exposure With Food Intake

Value	Food item							
	Rice	Egg	Chicken	Fish	Wheat	Vegetables	Pulses	Dairy
Correlation, *r*^a^	0.19	0.15	0.13	0.11	−0.12	0.03	0.03	−0.001
*P* value^b^	<.001	<.001	<.001	<.001	<.001	.30	.40	.97

^a^ Indicates bivariate correlation analysis.

^b^ Calculated using Bonferroni correction for multiple testing.

### Association of Arsenic Exposure With Cognition and Brain Structure and Function

**Figure 1.**
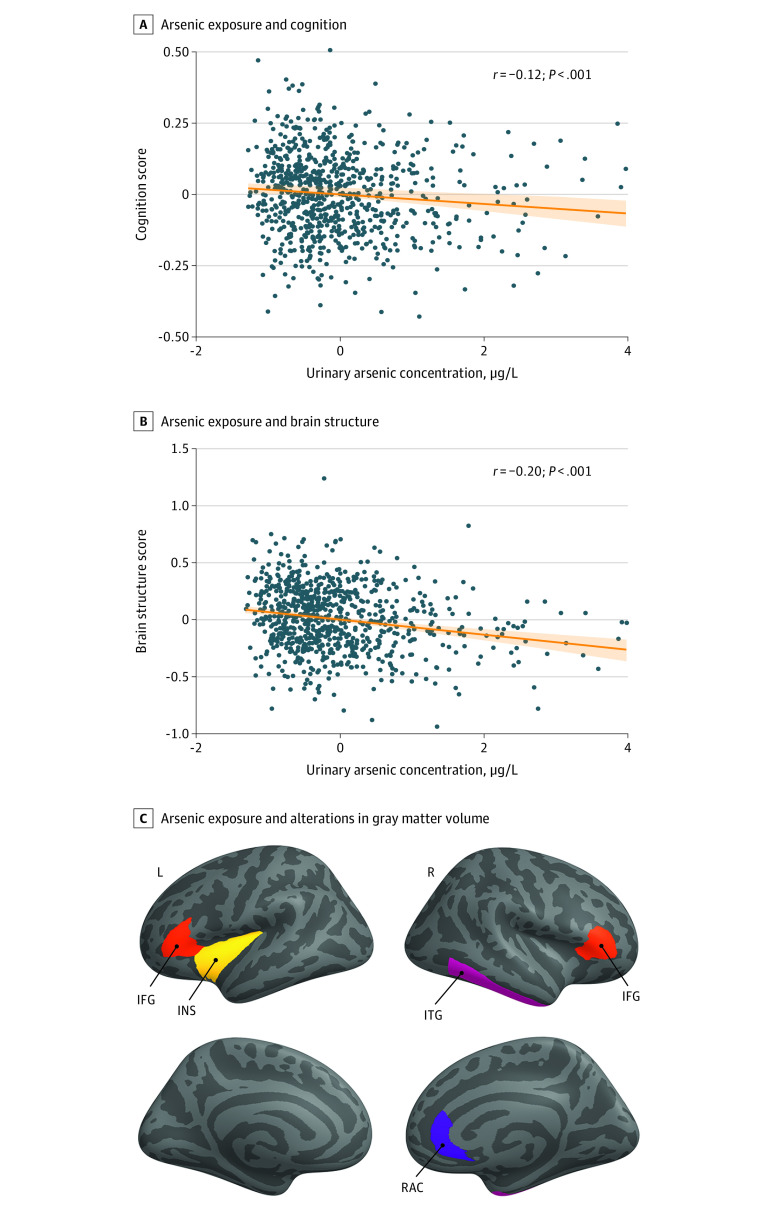
Association of Arsenic Exposure With Executive Function and Altered Gray Matter Volume A, B, Scatterplots show associations of arsenic exposure with cognition (A) and structural (B) factor scores derived from sparse-partial least squares analysis (to convert µg/L to µmol/L, multiply by 0.0133). Arsenic levels represent residues after covariate regression. The shaded areas in the plots indicate the CIs around the fitted values of the model. C, Brain regions show associations of arsenic exposure with gray matter volume. Colors are for visual representation of the brain areas only. IFG indicates inferior frontal gyrus; INS, insula; ITG, inferior temporal gyrus; L, left; R, right; RAC, rostral anterior cingulate.

Using sPLS, a method that maximizes covariance between data sets, we describe a cognitive syndrome related to low-level arsenic exposure at *r* = −0.12 (*P* = 5.4 × 10^−4^) (Figure 1A), characterized by associations in 5 of the 21 cognitive measures assessed, including attention (β = −0.05 [95% CI, −0.09 to −0.01]), working memory (β = −0.05 [95% CI, −0.08 to −0.01]), set shifting (β = −0.03 [95% CI, −0.07 to −0.004]), cognitive flexibility (β = −0.03 [95% CI, −0.06 to −0.003]), and risk taking (β = 0.06 [95% CI, 0.01-0.13). We conducted secondary analysis stratified by age and observed similar results for the association of arsenic with cognition across the 3 age bands for cVEDA: for 6 to 11 years, *r* = −0.12 (*P* = .04); for 12 to 17 years, *r* = −0.15 (*P* = .003); and for 18 to 23 years, *r* = −0.15 (*P* = .002).

We found alterations in gray matter volume (GMV) in a set of brain regions associated with arsenic exposure (*r* = −0.20 [*P* = 1.8 × 10^−8^]) (Figure 1B). These areas included the inferior frontal cortex (IFC; right pars triangularis: β = 0.13 [95% CI, 0.06-0.21]; left pars triangularis: β = 0.12 [95% CI, 0.04-0.21]), right inferior temporal cortex (ITC; β = −0.13 [95% CI, −0.22 to −0.04]), right rostral anterior cingulate (β = −0.09 [95% CI, −0.17 to −0.02]), and left insula (β = −0.11 [95% CI, −0.19 to −0.04]) (Figure 1C), areas that have been implicated in the cognitive symptoms identified.

**Figure 2.**
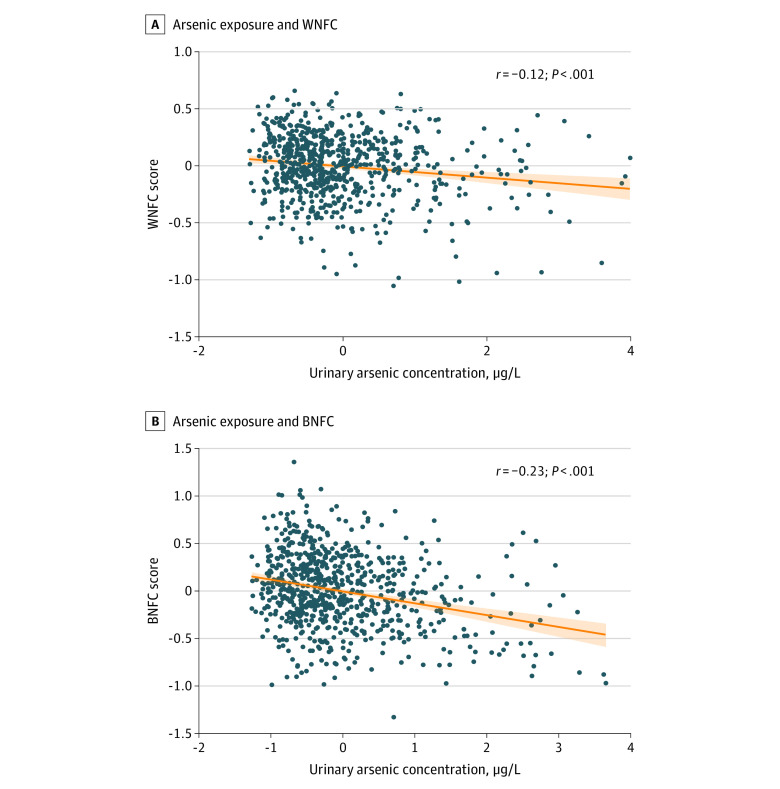
Association of Arsenic Exposure With Altered Brain Connectivity A, B, Scatterplots depict the association between arsenic exposure and within-network functional connectivity (WNFC) (A) and between-network functional connectivity (BNFC) (B) factor scores. The shaded areas in the plots indicate the CIs around the fitted values of the model. Scores were derived from sparse-partial least squares analysis. To convert µg/L to µmol/L, multiply by 0.0133. Arsenic levels represent residues after covariate regression.

We then examined the relation of arsenic exposure with resting state functional connectivity, measured among 53 independent components from NeuroMark (component labels are provided in eTable 2 in Supplement 1).^24^ Arsenic was associated with within-network functional connectivity (WNFC) at *r* = −0.12 (*P* = 7.5 × 10^−4^) (Figure 2A), characterized by altered connectivity in the cognitive domain within the right IFC (β = −0.12 [95% CI, −0.21 to −0.04]), middle frontal cortex (β = −0.01 [95% CI, −0.04 to −0.004]), and hippocampus (β = −0.09 [95% CI, −0.16 to −0.01]); in the visual domain at the middle occipital gyrus (β = −0.09 [95% CI, −0.18 to −0.01); and in the sensorimotor domain at the left postcentral gyrus (β = −0.02 [95% CI, −0.04 to −0.003) and paracentral lobule (β = −0.09 [95% CI, −0.18 to −0.005]) (eFigure 2A in Supplement 1).

Alterations in between-network functional connectivity (BNFC) associated with arsenic exposure were statistically significant at *r* = −0.23 (*P* = 1.8 × 10^−10^) (Figure 2B). Typically, BNFC for networks within a domain is positively correlated. We found a negative correlation of BNFC with arsenic exposure (eFigure 2B in Supplement 1). Negative associations were observed for (1) BNFC of left and right IFC (β = −0.09 [95% CI, −0.16 to −0.03) in the cognitive domain and (2) between the paracentral lobule and precentral gyrus (β = −0.11 [95% CI, −0.19 to −0.04]) in the sensorimotor domain. The arsenic exposure–associated reductions in the connectivity strength between these networks suggest less interaction between networks correlates with greater arsenic exposure.

Positive association between BNFC and arsenic levels was observed for (1) the cognitive domain and the default mode domain in the hippocampus with the posterior cingulate cortex (β = 0.09 [95% CI, 0.02-0.16]) and in the left inferior parietal lobule with the anterior cingulate cortex (ACC; β = 0.11 [95% CI, 0.03-0.19]); (2) the default mode domain and the visual domain in the posterior cingulate cortex with the middle temporal cortex (β = 0.11 [95% CI, 0.04 to 0.18]) and in the posterior cingulate cortex with the right middle occipital cortex (β = 0.11 [95% CI, 0.04-0.17]); (3) the cognitive domain and the visual domain in the middle frontal cortex with the fusiform gyrus (β = 0.14 [95% CI, 0.07-0.21]); (4) the subcortical domain and visual domain in the caudate and the right middle occipital cortex (β = 0.12 [95% CI, 0.04-0.21]); and (5) the default mode domain and the sensorimotor domain in the paracentral lobule with posterior cingulate cortex (β = 0.14 [95% CI, 0.07-0.23]). These associations delineate circuits through which affective and cognitive processes interact.

### Brain Mediator

To test whether the identified brain regions and networks mediate the associations between arsenic exposure and cognition, we conducted multivariate bias-corrected bootstrapped mediation analysis using the factor scores saved from sPLS. The unstandardized indirect effects observed for bootstrapped 95% CIs were statistically significant for structural substrate (*b* = −0.004 [95% CI, −0.007 to −0.002]) and WNFC (*b* = −0.004 [95% CI, −0.008 to −0.002]), but not for BNFC (*b* = −0.001 [95% CI, −0.004 to 0.003]) (Table 2). The proportion of mediation was 28.13% for structural substrate and 29.86% for WNFC. These brain areas (structure plus WNFC) may act as a neural endophenotype between arsenic and the manifestation of observable cognitive phenotypes (ie, executive function).

**Table 2.** Multiple Parallel Mediation Pathways Based on Bootstrapped Variability Around the Product of Nonstandardized Path Coefficient Estimates

Pathway	Estimate, *b* (95% CI)^a^			
	Brain structure	WNFC	BNFC	Cognitive phenotype
Direct effect				
Arsenic	−0.06 (−0.08 to −0.04)	−0.04 (−0.07 to −0.02)	−0.12 (−0.15 to −0.08)	−0.01 (−0.02 to 0.004)
Structural substrate	NA	NA	NA	0.06 (0.02 to 0.09)
WNFC	NA	NA	NA	0.09 (0.05 to 0.13)
BNFC	NA	NA	NA	−0.001 (−0.03 to 0.02)
Indirect effect				
Arsenic via structure	NA	NA	NA	−0.004 (−0.007 to −0.002)
Arsenic via WNFC	NA	NA	NA	−0.004 (−0.008 to −0.002)
Arsenic via BNFC	NA	NA	NA	0.0001 (−0.004 to 0.003)
Total	NA	NA	NA	−0.008 (−0.01 to −0.003)

Abbreviations: BNFC, between-network functional connectivity; NA, not applicable; WNFC, within-network functional connectivity.

^a^ Path coefficients based on 10 000 bootstrapped resamples with bias-corrected 95% CIs.

### Moderating Role of SES and BMI

Since the report by the United Nations Children’s Fund on arsenic contamination stated that the outcomes of arsenic exposure are exacerbated by poverty and poor nutrition,^1^ we used conditional process analysis to establish whether the identified mediation effects were conditional on the moderation of SES and BMI. The estimated regression coefficients are displayed in eTables 7 and 8 in Supplement 1. The bootstrapped 95% CIs for Index of Moderated Mediation suggested that the indirect effect of arsenic on cognition through structural substrate was moderated by both SES (*b* = 0.001 [95% CI, 0.002-0.004]) and BMI (*b* = 0.001 [95% CI, 0.001-0.003]). For the WNFC model, index of moderated mediation was not statistically significant for SES (*b* = 0.004 [95% CI, −0.002 to 0.003]) or BMI (*b* = 0.007 [95% CI, −0.001 to 0.003]). Thus, the outcomes of exposure to arsenic on brain structure and downstream executive function are exacerbated by poorer SES and lower BMI.

**Figure 3.**
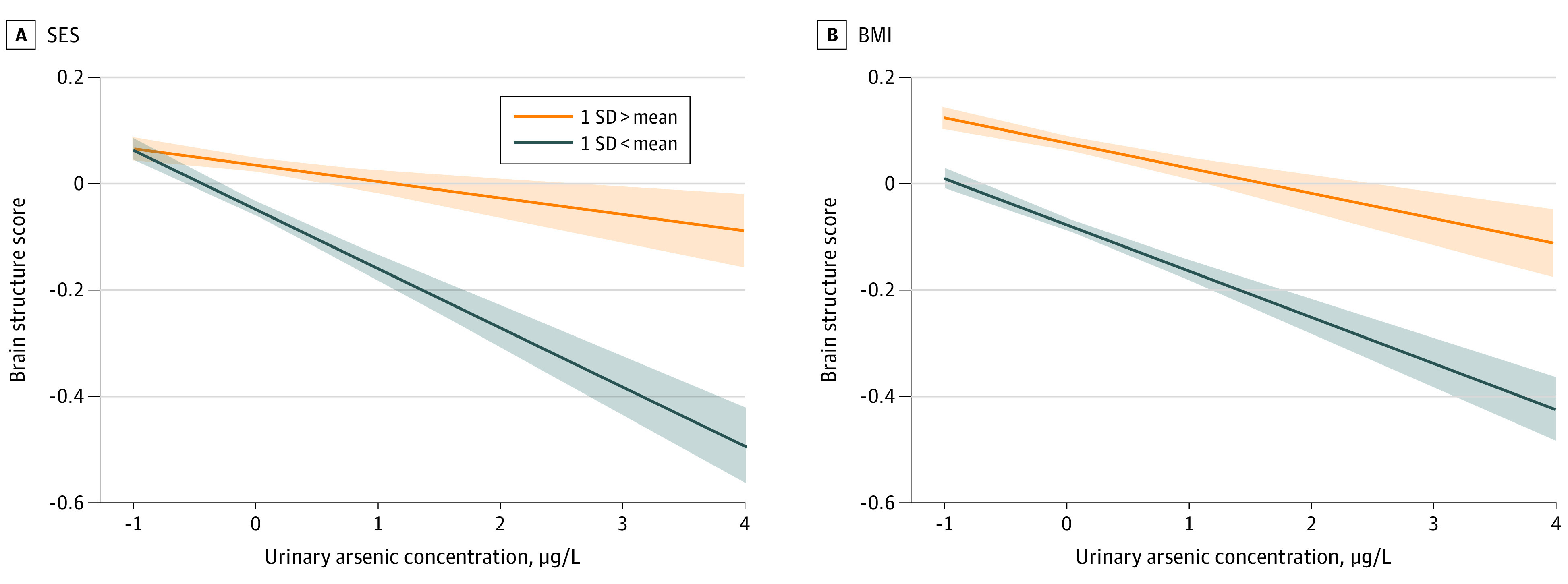
Association of Socioeconomic Status (SES) and Body Mass Index (BMI) With Arsenic Exposure Functions are graphed for 2 levels: high (ie, 1 SD above the mean) and low (ie, 1 SD below the mean). To convert µg/L to µmol/L, multiply by 0.0133. Arsenic levels represent residues after covariate regression. The shaded area in the plot indicates the confidence interval around the fitted values of the model (ie, the level of uncertainty around the estimate). The graph is for descriptive purposes only. All inferential analyses maintained continuous values.

For descriptive purposes, we plotted estimated structural substrate against arsenic. As highlighted in Figure 3A, for low SES (1 SD below mean) the effect of arsenic was stronger (*b* for simple slope = −0.10) than for higher (1 SD above mean) SES (*b* for simple slope = −0.03). Similarly, for BMI (Figure 3B), moderation effect was stronger for individuals with low BMI (*b* for simple slope = −0.08) than for individuals with high BMI (*b* for simple slope = −0.04).

## Discussion

In a population-based child, adolescent, and young adult sample in India, we describe a neurocognitive syndrome characterized by altered executive function that is associated with low-level arsenic exposure. This syndrome is characterized by cortical volumetric anomalies and disruptions in functional connectivity that mediate alterations in executive function, a group of cognitive processes involved in the allocation of attention and control over behavior, to meet adaptive goals.^25,26^ The cognitive processes associated with low-level arsenic exposure include deficits in attention, working memory, set shifting, cognitive flexibility, and risk-taking behavior. The set of brain regions mediating this association includes GMV of the IFC (pars triangularis) in the prefrontal region, the right ITC, right rostral ACC, and the left insular cortex. A further mediator consists of WNFC. Increased arsenic exposure is correlated with reduced WNFC in the cognitive domain, namely in the right IFC, middle frontal cortex, and hippocampus; the visual domain, namely in the middle occipital cortex; and the sensorimotor domain, namely in the left postcentral gyrus and paracentral lobule. While low-level arsenic exposure was associated with BNFC, no mediation was observed. Our results indicate that low levels of exposure to arsenic among participants residing in areas with reported groundwater arsenic content below World Health Organization thresholds^23^ are correlated with aberrations in structural and functional brain regions and alterations of cognitive processes of executive function.

The GMV and functional connectivity regions associated with arsenic exposure plausibly underline the cognitive alterations observed. Exposure to arsenic among children is of particular concern as exposure occurs during critical periods of neurodevelopment. The brain regions affected show only partial regional overlap, suggesting different levels of sensitivity to arsenic in different regions of the developing brain. One brain region where overlap is consistent is the bilateral IFC, including the pars triangularis, which contains the highest loadings within the GMV and WNFC sets. We found a positive association with GMV as well as negative associations with WNFC in the IFC and with BNFC in the cognitive domain for the bilateral IFC. These associations may reflect disruptive brain maturation and are linked with control of attention,^27,28^ set shifting,^29,30^ and risk taking,^31,32^ accounting for 3 of 5 arsenic-associated cognitive symptoms of executive function identified in our study, deficits of which underlie many psychiatric disorders in a transdiagnostic way.^33,34^ Interestingly, increased GMV of the IFC (Broca area) has been observed among children with developmental language disorder,^35^ a known symptom of arsenic exposure^17^ that was not assessed in this investigation. In the GMV set, we also found a negative association of arsenic exposure with left insula volume, right ACC volume, and right ITC. While we did not find an association with the salience network,^36^ of which the insula and ACC are a part, volume reduction in these regions has been associated with arsenic-related symptoms, namely impairments in attention^37,38^ and in risk taking.^39,40^ Reduction in GMV in the right ITC is associated with alterations in working memory,^41^ another cognitive process of executive function. In the WNFC set, we found further brain correlates of arsenic-associated working memory deficits, including reduced connectivity in the hippocampal network.^42,43^ The central hub of the arsenic-associated BNFC set, engaged in 5 BNFC networks, is the cingulate cortex, which is central to the default mode network (DMN), a network linked to a range of psychiatric disorders, including mood disorders and attention-deficient/hyperactivity disorder.^44,45^ We observed positive associations between the hippocampus and posterior cingulate connectivity, that is, the cognitive network and DMN. Another positive connection between these 2 networks was observed between inferior parietal and anterior cingulate connectivity. The DMN and cognitive network function in opposing direction (anticorrelation) in relation to attentional demands (ie, the DMN is active during rest while the cognitive network is active during task performance).^44,46^ Our findings suggest a disruption in the normal inverse association of the two in line with attention deficits.^44,47^ More generally, our observations suggest that arsenic exposure might be correlated with a delayed or disrupted maturation of large-scale networks, resulting in a compromised executive functioning, thus increasing general vulnerability for psychiatric disorders.^33,48^

While many studies have investigated at-risk populations in endemic areas of elevated arsenic levels in water, we observed participants living in areas with low arsenic water concentration.^23^ In our sample, exposure to arsenic was best correlated with food intake, indicating that background inorganic and organic arsenic in foods is a major contributor,^9,49^ which can spread arsenic exposure well beyond the areas of high risk. For example, we observed the highest correlation with rice intake, which is more commonly consumed in south India (Rishi Valley and Bangalore) and where highest mean arsenic levels were observed. Thus, chronic exposure to arsenic could be creating a “silent pandemic” affecting large portions of the global population.^50^ Importantly, arsenic exposure may be an easily modifiable risk factor: while treatment of water is possible but complicated and costly, cooking rice in a certain way removes the naturally occurring arsenic in brown rice by over 50% and in white rice by 74%, while not reducing micronutrients in the rice.^51^

We found that SES and BMI moderated the association among arsenic, brain structure, and executive function. Our results show that children with lower standards of living and low BMI might be more vulnerable to environmental insults associated with arsenic and might point toward a syndemic relation resulting in greater health problems. The consequences of SES disparity are evident in both executive function and brain structure. Thus, children from high SES families may be relatively protected from arsenic-associated brain and cognitive deficits. Further, the stronger association among arsenic, brain, and cognition in children with low BMI suggest that reductions in environmental insults associated with arsenic might more greatly benefit children experiencing higher overall environmental adversity.

Our results corroborate the circumstances facing children in India with lower SES. The disparity in experience or exposure such as family stress, cognitive stimulation, or corresponding environment such as housing, neighborhood crime, or parental education associated with poverty could possibly underly the moderation observed. Further, low-income populations are more likely to consume unsafe drinking water or food products that are not processed to remove arsenic content. The nutritional status could be a result of poverty or poor food choices and could reflect the level of sensitivity toward arsenic metabolism. Undernutrition in India is a public health problem, and the National Family Health Survey^52^ has identified 38% of Indian children as having stunted growth and 36% as underweight. The lower BMI observed in the subset of arsenic-exposed children and adolescents could be a component of the prevailing state of child undernutrition in India or an association of possible long-term arsenic exposure. Either way, our findings are suggestive of a wider public health problem associated with nutrition and living conditions among children in India compounded by environmental arsenic exposure that ultimately affects brain development and cognition.

Our findings have public health implications that highlight the importance of strategies to minimize the adverse effects of arsenic. The cVEDA is an observational cohort and the results provide evidence of the effect of arsenic in a large sample that was recruited at a population level without conditioning on the risk being studied. The multisite coverage further adds to the robustness of the results. Next, we have characterized the effects of low levels of arsenic on brain structure and function among children and adolescents. The biological and clinical underpinning of arsenic exposure expands our understanding of its impact on cognition. Additionally, factors such as BMI and SES are important from policy viewpoints and interventions to improve living environment may have the potential for a large public health benefit.

### Limitations

This study has some limitations. Causality cannot be inferred because of the cross-sectional, observational nature of the cVEDA study’s baseline data. Further, risk must be understood in the data’s psychosocial context. Many factors account for variance in brain and subsequent cognitive functioning. However, by elucidating associations with arsenic, we may be better able to identify precise environmental factors to serve as targets for change. These associations were observed for the cohort, and future studies should examine the association at different age points. Additionally, the urinary arsenic measure used in the study, although commonly used in epidemiological studies, is subject to daily variations. It is important to note that in India, with increasing urbanization and uneven regulatory compliance, the general population is exposed to environmental pollution at different risk levels. Therefore, we assume that the exposure to risk is constant. Further, as all environmental pollutants are toxic chemicals, we conclude that any exposure is harmful, which in this study is measured at cross-sectional time points. Thus, we are focusing on low-grade exposure, as is most likely to be experienced by most of the general population, over a period of time.

## Conclusion

The findings of this cross-sectional study suggest that low-level exposure to arsenic was associated with alterations in executive functioning and the underlying structural and functional brain substrate in developing children and adolescents. The syndrome identified might affect a significant proportion of the world population either through direct exposure in endemic areas or through distribution of food contaminated with trace amounts of arsenic that travels well beyond its original source. These deficits in executive function, while mild and not very prominent at an individual level, could nonetheless be indicative of vulnerability to future psychopathology, and might also result in significant adverse consequences on a population level, contributing to an overall increase in school failures, diminished economic productivity, and increased risk of criminal and antisocial behavior.^50^ We have further described modifiable environmental risk factors, including SES and BMI, that moderate the risk for arsenic-associated adversity, thus pointing toward a way to reduce risk and alleviate brain and cognitive deficits related to low-level arsenic exposure.

## Notes

Supplement 1. **eMethods 1.** cVEDA Cohort and Data **eTable 1.** Acquisition Parameters Across cVEDA Sites **eTable 2.** Component Labels Extracted Using NeuroMark **eFigure 1.** Study Sites and Flowchart Illustrating Sample Used in This Analysis **eMethods 2.** Statistical Analysis **eTable 3.** Demographics of the cVEDA Cohort **eTable 4.** Demographics of Participants With and Without Cognitive Data **eTable 5.** Urine Arsenic Levels (μg/L) Among Participants by Site **eTable 6.** Comparison of Total Urinary Arsenic (μg/L) **eFigure 2.** Urinary Arsenic Associated With Altered Brain Connectivity **eTable 7.** Conditional Indirect Effect—Socioeconomic Status **eTable 8.** Conditional Indirect Effect—Body Mass Index **eMethods 3.** Sensitivity Analysis **eTable 9.** Correlation Values With Significance Estimating the Relationship of Arsenic With Physical Environment **eTable 10.** Mediation Pathways Based on Bootstrapped Variability Around the Product of Nonstandardized Path Coefficient Estimates for Structural Substrate **eTable 11.** Mediation Pathways Based on Bootstrapped Variability Around the Product of Nonstandardized Path Coefficient Estimates for WNFC
eReferences

Supplement 2.
Nonauthor Collaborators

Supplement 3.
Data Sharing Statement
