# Error in an Affiliation and Corresponding Author Address

**DOI:** 10.1001/jamanetworkopen.2023.21313

**Published:** 2023-06-13

In the Original Investigation “Neurocognitive Analysis of Low-level Arsenic Exposure and Executive Function Mediated by Brain Anomalies Among Children, Adolescents, and Young Adults in India,”^1^ published May 12, 2023, the name of an author affiliation, the Indian Council of Medical Research–Centre on Noncommunicable Diseases, changed after submission of the article. The amended affiliation now reads, “Indian Council of Medical Research–Centre for Ageing and Mental Health, Kolkata, India.” This also affected the address of co–corresponding author Amit Chakrabarti, MD, which has been updated. This article has been corrected.^1^
