# Supplementary material for: Neurocognitive Analysis of Low-level Arsenic Exposure and Executive Function Mediated by Brain Anomalies Among Children, Adolescents, and Young Adults in India
Source: JAMA Netw Open. 2023 May 12;6(5):e2312810. doi: 10.1001/jamanetworkopen.2023.12810 (PMC10182429; doi:10.1001/jamanetworkopen.2023.12810)
Supplement: Supplement 1. — eMethods 1. cVEDA Cohort and Data eTable 1. Acquisition Parameters Across cVEDA Sites eTable 2. Component Labels Extracted Using NeuroMark eFigure 1. Study Sites and Flowchart Illustrating Sample Used in This Analysis eMethods 2. Statistical Analysis eTable 3. Demographics of the cVEDA Cohort eTable 4. Demographics of Participants With and Without Cognitive Data eTable 5. Urine Arsenic Levels (μg/L) Among Participants by Site eTable 6. Comparison of Total Urinary Arsenic (μg/L) eFigure 2. Urinary Arsenic Associated With Altered Brain Connectivity eTable 7. Conditional Indirect Effect—Socioeconomic Status eTable 8. Conditional Indirect Effect—Body Mass Index eMethods 3. Sensitivity Analysis eTable 9. Correlation Values With Significance Estimating the Relationship of Arsenic With Physical Environment eTable 10. Mediation Pathways Based on Bootstrapped Variability Around the Product of Nonstandardized Path Coefficient Estimates for Structural Substrate eTable 11. Mediation Pathways Based on Bootstrapped Variability Around the Product of Nonstandardized Path Coefficient Estimates for WNFC eReferences [file jamanetwopen-e2312810-s001.pdf]

Supplementary Online Content

Vaidya N, Holla B, Heron J, et al; Consortium on Vulnerability to Externalizing Disorders and Addictions (cVEDA). Neurocognitive analysis of low-level arsenic exposure and executive function mediated by brain anomalies among children, adolescents, and young adults in India. *JAMA Netw Open*. 2023;6(5):e2312810. doi:10.1001/jamanetworkopen.2023.12810

eMethods 1. cVEDA Cohort and Data

eTable 1. Acquisition Parameters Across cVEDA Sites

eTable 2. Component Labels Extracted Using NeuroMark

eFigure 1. Study Sites and Flowchart Illustrating Sample Used in This Analysis

eMethods 2. Statistical Analysis

eTable 3. Demographics of the cVEDA Cohort

eTable 4. Demographics of Participants With and Without Cognitive Data

eTable 5. Urine Arsenic Levels (µg/L) Among Participants by Site

eTable 6. Comparison of Total Urinary Arsenic (µg/L)

eFigure 2. Urinary Arsenic Associated With Altered Brain Connectivity

eTable 7. Conditional Indirect Effect—Socioeconomic Status

eTable 8. Conditional Indirect Effect—Body Mass Index

eMethods 3. Sensitivity Analysis

eTable 9. Correlation Values With Significance Estimating the Relationship of Arsenic With Physical Environment

eTable 10. Mediation Pathways Based on Bootstrapped Variability Around the Product of Nonstandardized Path Coefficient Estimates for Structural Substrate

eTable 11. Mediation Pathways Based on Bootstrapped Variability Around the Product of Nonstandardized Path Coefficient Estimates for WNFC

eReferences

This supplementary material has been provided by the authors to give readers additional information about their work.

© 2023 Vaidya N et al. *JAMA Network Open*.

eMethods. cVEDA Cohort and Data

The cVEDA cohort: cVEDA, a large neurodevelopmental cohort in India, was set up to enable the long-term study of the developmental trajectories of brain and psychological functioning, and the effects of exposome and genome in modulating these trajectories, to influence vulnerability to psychiatric disorders. Participants were recruited from seven sites representing five different geographical locations in India, of all gender, ethnicity, socio-cultural strata's and urban-rural living. The study involves a thorough assessment of behavioural, neuropsychological, clinical, and environmental exposures of each subject. Participants also undergo biological characterization with collection of T1 weighted MRI, diffusion tensor imaging, resting state functional MRI and genetics data including blood and urine for assay of neurotoxin exposures.[1,2] cVEDA study was approved by the Health Ministry Screening Committee, Ministry of Health and Family Welfare, Government of India. The study protocol was approved by the Institutional Ethics Review Boards of National Institute of Mental Health and Neurosciences (NIMHANS) Bangalore, India (Item No. VII, SI. No. 7.08, Behavioural Sciences) and all other regional collaborating institutions. Written informed consent was obtained from participants over the age of 18 and from parents of participants under 18 years of age (along with assent from minor participants). The study was conducted in accordance with the Declaration of Helsinki (1964 and later versions).

cVEDA consists of seven study recruitment centres and corresponding catchment areas including: Imphal (Manipur); Asansol (West Bengal); Mysore (Karnataka); National Institute of Mental Health and Neurosciences [NIMHANS] Bangalore (Karnataka); Post Graduate Institute of Medical Education and Research (PGIMER) (Chandigarh, Punjab and Haryana); Rishi Valley (Madanapelle, Andhra Pradesh); Saint John's Research institute [SJRI], Bangalore (Karnataka). The target sample sizes at each recruitment centre were decided based

on the capacity to recruit individuals over a 3-year recruitment period (2016-2019), given the site lead's understanding about ground realities and experience from past studies. The same represents five geographically, ethnically, and socio-culturally distinct regions including a variety of environmental risks: toxic exposures (coalmines), slum-dwellers, socio-political conflict zones and urban and rural areas. Two to five recruiters per site approached participants at or in the vicinity of the study site, and research purposes and involvement were explained to both the parent(s) and child/adolescent. Full details on recruitment catchment areas and inclusion and exclusion criteria have been previously published in protocol paper and cohort profile.[1,2]

In this sub-study we use cross sectional data from this observation cohort that followed a non-probabilistic convenience sampling. We did not condition on risk factors for selection into the sample. Exclusion criteria included legal blindness/deafness, seizure disorder active in the last one month, severe physical or active mental illness, refusal of consent, or inability to participate in follow-up assessments. Individuals with specific contra-indications (metal implants, electrical devices, severe claustrophobia) were excluded from neuroimaging. All neuroimaging participants also underwent urine arsenic measurement and were included in this sub-study. Our data came from the cVEDA 1.4 release, for the variables of interest there were complete data for 1014 participants. This study followed the Strengthening the Reporting of Observational Studies in Epidemiology (STROBE) reporting guideline.

Urinary Arsenic: The current analyses include a subgroup of the sample (n=1014) for whom arsenic measurement was carried out. Mid-stream urine samples (25 ml) were collected in clean and capped polythene bottles, stored and brought to the laboratory at 4°C and preserved in deep fridge at -20° C until analysis. Urine samples for arsenic measurement were processed by pre-analysis treatment of the samples with ammonium peroxo sulphate followed by microwave

© 2023 Vaidya N et al. *JAMA Network Open*.

digestion as described by Sysalova et. al.[3] in high pressure vessel with excess fluoride ion. Measurement was performed using an atomic absorption spectrometer with flow injection hydride generator system (PerkinElmer AA800, USA) and Zeeman-effect background correction.[4] The parameters used in atomic absorption spectrometer (PerkinElmer AA800) to analyse urinary arsenic were slit width 0.7 nm, wavelength 193.7 nm, quartz tube temperature 900 degree C, amp current 380 mA, lamp energy 48, carrier solution HCl 10% (v/v), reducing agent NaBH4 0.2% (w/v) in 0.05% (w/v) NaOH, purging gas (argon) flow rate 70 ml / min and sample loop 500ul. For quality assurance and to validate analytical procedure on each day of analysis, certified arsenic reference material (Certipur, Millipore, USA) comparable to NIST Standard Reference Material (National Institute of Standards & Technology, Gaithersburg, MD, USA) was used. The recorded concentrations of the reference material were within the recommended range. 10% HCl solution (v/v) was used as procedural blank and 10% random samples were run in duplicates. Spiked recovery rate was close to 100%. The limit of detection was 0.26 ug/L.

Dietary intake: Short Food Questionnaire (SFQ)[5] measures dietary intake. A list of different food items with frequency response is presented to participant, who reports how often each item is consumed in a week. The frequency of intake was used for analysis in the present study.

Cognition: Neuropsychological battery was administered using tablet devices via Psytools software (Delosis Ltd, London, UK), by trained psychologists. The tests capture versatility to monitor function over time for longitudinal study, brevity to ensure low respondent burnout, validity, and reliability for our state of the art accelerated longitudinal design. All tasks were non-verbal, the testing conditions were kept constant, and we used computerized tasks for clear visual stimuli, large target areas, simple task construction and step by step instruction. The analyses included performances on:

© 2023 Vaidya N et al. *JAMA Network Open*.

1. Balloon analogue risk task (BART): The BART is presented as a computer-based measure of risk-taking propensity. In this task, participants are presented with 90 balloons, one at a time. They pump the balloon to increase its size and its monetary value. At any point, they can stop pumping to collect the amount earned. However, if the balloon explodes while pumping, the amount is lost, and they move to the next balloon. The explosion point varies across balloons and participants are required to determine how much to pump up the balloon. The primary dependent measure on the BART is the adjusted number of pumps across balloons.[6]

2. CORSI: Corsi block tapping task is used to measure visuospatial attention and working memory.[7,8] Participants are presented with nine blue buttons on the screen. On each trial, the buttons lit up one at a time in a certain order. Participants are required to remember this order and click on each button in the same sequence. The trial starts with a sequence of two buttons, and they get two attempts at each length. The sequence increases by one when they get at least one of the two trials correct, up to a maximum sequence of 9 buttons. Next, participants repeat the trial, but backwards, i.e., they trace the sequence in the reverse order. The length of the highest correctly entered sequence is recorded as corsi span forwards, and backwards, respectively.

3. Digit Span Test (DST): The DST is common among most research on cognitive functioning and is used to test short-term memory, i.e., immediate recall abilities and attention.[9] Participants are presented with a sequence of digits, one at a time on the screen. Each digit occurs only once during a list. Participants are then asked to type the list of digits exactly in the same order. They start with a list of two items and get up to three different lists at each length. If they recall two out of three lists completely and correctly, they move to the next length (maximum up to 9 digits). Next, the participants

© 2023 Vaidya N et al. *JAMA Network Open*.

are asked to type the digits in reverse order. Performance is reported as span score, i.e., the maximum length of digits correctly typed. Forward span captures attention capacity. Backward span is an executive task particularly dependent on working memory.

4. Emotional Recognition Task (ERT): ERT measures the ability to identify emotions in facial expressions. Each face appears for 200ms and participant is asked to select the appropriate emotion from happy, sad, angry, disgust, surprise, fear and neutral. The outcome measures percentage of correctly labelled emotions, and overall response latencies, for individual emotion groups and for all emotions together.[1]

5. Now or Later: The 27-item questionnaire assesses discounting of hypothetical monetary amounts over time and across three different delayed-reward magnitudes (small, medium, or large). Within each reward-magnitude category, the nine choices define 10 ranges of discount rates. Participants are asked to choose between a smaller, immediate reward or a larger delayed reward and are assigned representative k-value based on their response that corresponds to the range of discount rates, within each reward magnitude. Delayed discounting a measure of temporal discounting, is one element that underlines decision making.[10] It is calculated by computing geometric mean of the three rates for the reward magnitudes.

6. Social Cognition Rating Tools in the Indian Setting (SOCRATIS): SOCRATIS is used to measure theory of mind (TOM) and social perception.[11] Participants are shown first order, second order, metaphor-irony, and faux pas recognition stories. These stories are followed by questions to examine, at different levels of complexity, the participants ability to infer mental states of the characters in these stories. Three indices are derived based on the proportion of correct responses made. 1st order TOM is scored on two 1st

© 2023 Vaidya N et al. *JAMA Network Open*.

order stories and the metaphor stories, 2nd order TOM is scored on two 2nd order stories, and the irony stories and faux pas recognition index is based on faux pas detection and non-faux pas rejection.

7. Stop Signal Task (SST): SST derived from the task of Logan and Cowan.[12] Participants are instructed to respond to an imperative go signal, i.e., press right or left arrow when an image of the same appears on the screen. When an additional stop signal occurs, i.e., the arrows are followed by an additional upward arrow, participants are instructed to not respond (inhibit reaction). SST measures response inhibition calculated by subtracting average stop signal delay from the probability of responding on stop trials as a function of stop signal delay [p(respond/signal)].

8. Trail making test (TMT): The test consists of 3 smaller tasks. In the first task participants are presented with 20 dots. As they click the dot which is lighted the light shifts to another dot. Participants track this shift in light as quickly as they can. Task 2 of the test requires participants to connect the dots in alphabetical order and task 3, which is more complex, requires an alternation between numbers and alphabets (1, A, 2, B, 3, C, etc.). TMT measures participant's executive ability, i.e. set shifting calculated as the ratio of the time taken on task 3 and that on task 2.[13]

9. Card sorting test: The Berg Card Sorting Test, which is more commonly known as the Wisconsin Card Sorting Test, is a measure of executive function, specifically, cognitive flexibility.[14] The computer presents the participant with four key cards, which contain shapes that differ in geometric type, colour, or number of shapes. The participant is required to correctly match a given card to one of four displayed cards through trial and error using a sorting rule based on either type, colour, or number. The participant receives feedback indicating whether the choice is correct or incorrect. After 10 correct

© 2023 Vaidya N et al. *JAMA Network Open*.

trials, the sorting rule changes without notice, and the participant, has to figure out the sorting strategy for the newly presented cards. Scoring includes performance on categories achieved, trials, errors, and perseverative errors.

MRI acquisition and pre-processing: Participants from all sites were scanned at five different 3-Tesla MRI scanners across India. Participants from 3 sites (NIMHANS, SJRI, and RVRHC) were scanned at the Bengaluru site at NIMHANS on two MRI scanners (Siemens Skyra and Philips Ingenia). The other MRI sites were at Chandigarh (Siemens Verio), Mysore (Philips Ingenia) and Kolkata (Siemens Verio). For this study, we used the baseline T1w structural MRI and resting state functional MRI scans. To ensure comparability of image-acquisition techniques and 'pool' ability of the multi-site MRI data, a set of parameters, particularly those directly affecting image contrast or signal-to-noise were held constant across sites based on ADNI protocol (http://www.loni.ucla.edu/ADNI/Cores/index.shtml). Full technical specifications are available for download at http://cveda-project.org/standard-operating-procedures/ and technical parameters are detailed in eTable 1.

eTable 1. Acquisition Parameters Across cVEDA Sites

Site	Scanner Model	dx (mm)	dy (mm)	dz (mm)	TR (ms)	TE (ms)	FA	Matrix Size (mm)	Sag slices
Bengaluru	Ingenia	1·0	1	1	6·5	2·9	9	256 x 256	211
Bengaluru	Skyra	1·2	1	1	2300	3	9	256 x 240	176
Chandigarh	Verio	1·2	0·5	0·5	2300	3	9	512 x 240	176
Kolkata	Trio	1·2	1	1	2300	3	9	256 x 240	176
Mysuru	Ingenia	1·2	1	1	6·9	3·2	9	256 x 256	170

Abbreviations: dx, dy, dz are voxel dimensions; TR, repetition time; TE, echo time; FA, flip angle; Sag Slices, number of sagittal slices. (Note that TR is defined differently between manufacturers).

T1 weighted MRI: Visual inspection of image quality was performed prior to each data release and standardized pre-processing was also applied. A detailed QC and pre-processing SOP is available at the cVEDA website. In brief, visual inspection was performed to exclude low-quality images (movement artefacts, brace artefacts and field inhomogeneities, etc.). AFNI's

“fat proc axialize anat” function was used for AC-PC alignment. The datasets were resampled (using a high order sinc function to minimize smoothing) to a 1mm isotropic voxel size. Brain volumes were estimated from the T1-weighted MRIs using the FreeSurfer software suite (FS Stable v6.0),[15] a set of automated tools for surface-based reconstruction of the brain. Using Freesurfer’s automated gyral-based labelling, a set of 68 cortical gray-matter brain regional volumes were extracted (34 in each hemisphere) based on the Desikan-Killiany atlas.[16]

Resting state fMRI: The functional data was motion corrected by applying a rigid body registration of each volume to the middle volume (FSL MCFLIRT). This was followed by the slice-timing correction to account for timing difference in slice acquisition. Non-brain tissue was removed (FSL BET) and coregistered to high-resolution T1 image (FSL FLIRT using the BBR algorithm). Motion correcting transformations, BOLD-to-T1w transformation and T1w-to-template (MNI) warp were concatenated and applied in a single step using Advanced Normalization Toolbox (ANTs v2.1.0) using Lanczos interpolation. Frame-wise displacement was calculated for each functional run and ICA-based Automatic Removal Of Motion Artifacts (AROMA) was used to generate non-aggressively denoised data. Lastly, the denoised data were resampled to 2mm isotropic and smoothed using a 4mm non-linear filter using FSL SUSAN. The NeuroMark automatic independent component analysis (ICA) pipeline which uses previously derived component maps as priors for spatially constrained ICA was used to derive 53 ICs from rsfMRI based on Multiple-Objective Optimization (MOO).[17] NeuroMark is a reliable ICA-based pipeline that automatically estimates functional regions adaptable to each individual subject and comparable across subjects by taking advantage of the reliable brain network templates extracted from 1828 healthy controls as guidance. Two large healthy datasets, i.e., the human connectome project (HCP, http://www.humanconnectomeproject.org/) and the genomics superstruct project

© 2023 Vaidya N et al. *JAMA Network Open*.

(GSP, https://dataverse.harvard.edu/dataverse/GSP), were used for the construction of the templates. Group ICA was performed on the GSP and HCP datasets, respectively, and the identified independent components (ICs) from the two datasets were then matched by comparing their group-level spatial maps. The reproducible IC pairs were further evaluated by examining their peak activations and low-frequency fluctuations of their corresponding TCs. ICs were defined based on prior anatomical and functional knowledge (validated by 5 fMRI experts). These were then used as references to calculate spatial maps for the dataset in the present study. The ICs as reported by NeuroMark, are shown in eTable 2 and are arranged into seven functional domains: subcortical (SC), auditory (AUD), visual (VS), sensorimotor (SM), cognitive-control (CC), default-mode (DM), and cerebellar (CB) domains. For each of the ICs, a participant-specific spatial component and time-course were generated by back reconstruction methods. Each voxel within the participant-specific spatial component represents the coherence of the time courses between the given voxel and the participant-specific time-series. The tmap statistics (t > mean + 4*std) were used to compute default group-level mask for each IC and the average of the coherence values for all voxels within each component at the group-level mask was defined within-network functional connectivity (WNFC). The correlation of the time courses between any two of the participant-specific components were defined as between-network functional connectivity (BNFC).

eTable 2. Component Labels Extracted Using NeuroMark

**Component name**	**Peak Coordinate (mm)**		
Sub Cortical Domain (SC)			
Caudate	6	4	10
Subthalamus/hypothalamus	0	-22	-5
Putamen	26	2	-10
Caudate	24	6	-10
Thalamus	0	-22	10
Auditory Domain			
Superior temporal gyrus [STG]	66	-22	15
Middle temporal gyrus [MTG]	60	2	5
Sensorimotor Domain			
Postcentral gyrus [PoCG]	60	-4	25

© 2023 Vaidya N et al. *JAMA Network Open*.

Left postcentral gyrus [L PoCG]	-40	-22	65
Paracentral lobule [ParaCL]	0	-24	65
Right postcentral gyrus [R PoCG]	42	-20	65
Superior parietal lobule [SPL]	24	-46	70
Paracentral lobule [ParaCL]	28	-8	55
Precentral gyrus [PreCG]	0	0	65
Superior parietal lobule [SPL]	-18	-76	55
Postcentral gyrus [PoCG]	54	-26	55
Visual Domanin			
Calcarine gyrus [CalcarineG]	0	-82	35
Middle occipital gyrus [MOG]	-24	-96	5
Middle temporal gyrus [MTG]	54	-64	10
Cuneus	0	-86	15
Right middle occipital gyrus [R MOG]	30	-72	-15
Fusiform gyrus	30	-46	-10
Inferior occipital gyrus [IOG]	-25	90	-25
Lingual gyrus [LingualG]	0	-84	0
Middle temporal gyrus [MTG]	-50	-66	-5
Cognitive Control Domain			
Inferior parietal lobule [IPL]	42	-64	55
Insula	46	18	-5
Superior medial frontal gyrus [SMFG]	0	56	25
Inferior frontal gyrus [IFG]	-52	20	-5
Right inferior frontal gyrus [R IFG]	54	20	-5
Middle frontal gyrus [MiFG]	54	18	35
Inferior parietal lobule [IPL]	-48	-54	55
Left inferior parietal lobue [R IPL]	48	-40	60
Supplementary motor area [SMA]	-52	20	-5
Superior frontal gyrus [SFG]	-26	26	55
Middle frontal gyrus [MiFG]	35	52	30
Hippocampus [HiPP]	16	-4	-15
Left inferior parietal lobue [L IPL]	-54	-34	55
Middle cingulate cortex [MCC]	0	24	40
Inferior frontal gyrus [IFG]	46	48	5
Middle frontal gyrus [MiFG]	-30	62	10
Hippocampus [HiPP]	0	-42	-30
Default mode domain			
Precuneus	0	-76	40
Precuneus	6	-46	5
Anterior cingulate cortex [ACC]	0	54	0
Posterior cingulate cortex [PCC]	0	-28	30
Anterior cingulate cortex [ACC]	0	12	-10
Precuneus	0	-64	55
Posterior cingulate cortex [PCC]	0	-66	35
Cerebellar Domain			
Cerebellum	-36	-60	-40
Cerebellum	-6	-82	-25
Cerebellum	0	-46	-40
Cerebellum	36	-70	-40

Moderators:

Body Mass index: BMI was derived from height and weight measurements. Height was measures using standard protocol to the last complete centimetre; weight was measured to the nearest 100g. BMI was calculated as weight (kg)/height (m^2).

© 2023 Vaidya N et al. *JAMA Network Open*.

Socio-economic status: SES was operationalized by standard of living index, which is a comprehensive and commonly used measure of SES, reflective of the diversities in economic and social standing in Indian system. It was calculated by adding scores assigned to various items, based on the national family health survey.[18] The items are categorized into house type, kitchen facility, toilet facility, source of lighting, source of cooking, source of drinking water, ownership of house, ownership of agricultural/irrigated land, ownership of livestock and ownership of durable goods/family assets. Total score represents standard of living index of the household, i.e., socio-economic status.

Study sample

The cVEDA sub sample used in this analysis were individuals who had undergone MRI scans. MRI scans that did not pass Quality Control (QC) due to low-quality images (movement artefacts, brace artefacts and field inhomogeneities, etc.) were excluded, which is a common approach in neuroimaging studies. Participants with and without cognitive data did not differ, as shown below (eTable 3). We used complete-cases analysis approach for the study.

eFigure 1. Study Sites and Flowchart Illustrating Sample Used in This Analysis

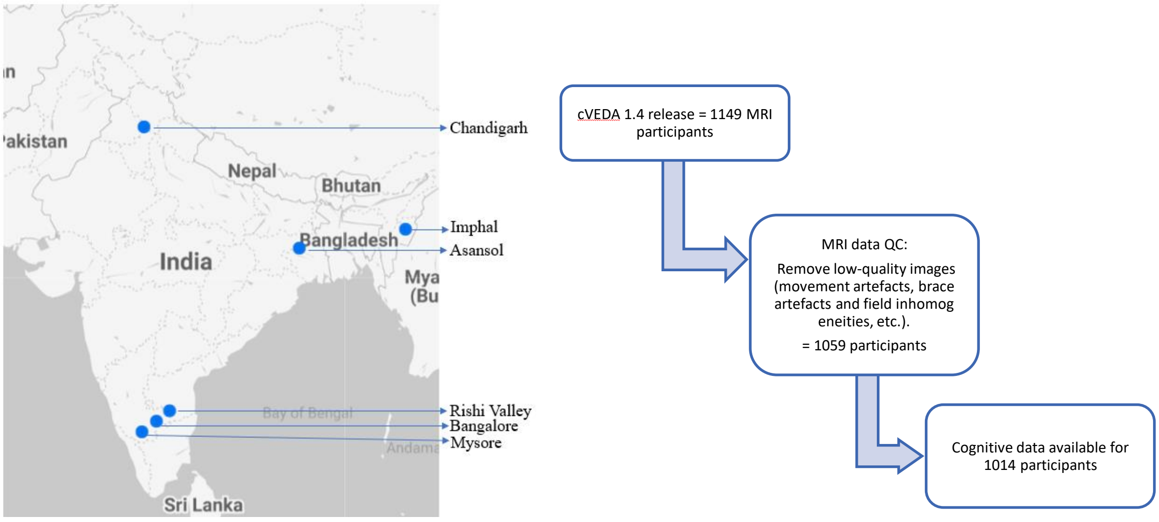

© 2023 Vaidya N et al. *JAMA Network Open*.

eMethods 2. Statistical Analysis

We used Sparse Partial Least Square (sPLS) analysis to determine the relationship between arsenic, cognition and brain structures and functions. In standard PLS,[19] cofounders are normally dealt with by regressing covariates on different data matrixes and applying the PLS algorithm to the residuals. We identified age, sex, site, education level and total intracranial volume as covariates. Data were regressed on these covariates and residual data matrixes were used for the next steps. Further in PLS, all variables contribute to associations between data and therefore to understand which specific features result in association, sparsity was induced by setting some PLS weights to zero through the application of L_1 penalization.[20] L_1 penalty forces weights that make negligible contribution to the variance explained to zero, thus, only most important variables that contribute to the relationship are selected. Although L_1 penalization is widely used, it has been shown to be unstable for feature selection for large number of variables specially if they are multicollinear, as in this investigation. Stability selection is used to address this, where data are repeatedly resampled and variables selected in 90% of the resamples are taken for final model, thus, variables exhibiting real effect are selected rather than noise. We applied stability selection, using hundred resampling procedures, as suggested by Meinhausen et al.,[21] to ensure that features retained are robustly associated with arsenic. Sparsity values was set at a random between 1 and $\sqrt{p}$, where p is the number of features in the relevant data. Thus, variables were stable across subjects (resampling) as well as different penalty thresholds. sPLS method thereby carried out dimension reduction and variable selection simultaneously. The sPLS approach used here is non-parametric in nature, therefore we used ten-fold cross-validation to quantify the strength and significance of the associations of individual brain and cognitive measures with arsenic. Bootstrapped confidence intervals (10,000 times) for the coefficients of the selected predictors were obtained. We

© 2023 Vaidya N et al. *JAMA Network Open*.

replicated this analysis five times to assess the stability of the estimates, i.e., ensure CI are consistent. Model significance was ascribed using a permutation testing procedure.

To further gauge the degree to which the brain factor identified by sPLS may act as an intermediary endophenotype between arsenic and selected cognitive phenotypes, we conducted mediation analysis. The mediation pathways were defined by the product term of the pathway of interest. Because standard errors underlying mediation pathway (i.e. the product terms) are known to be unreliable, we bootstrapped all indirect effects 10,000 times with bias corrected confidence intervals (95%). The mediation pathways are thus based on the bootstrapped variability around the product of non-standardized path coefficient estimates (i.e., *b*).

To provide a conceptual overview of the patterns in the data as well as context for the relationships, we conducted further analysis using data on participant's socio-economic status and body mass index. Moderation was assessed by calculating the index of moderated mediation suggested by Hayes.[22] The conditional effect of the variables and bias-corrected confidence interval using 10,000 bootstrapped resamples were generated. Indirect effects were estimated at different levels of the moderator according to the criteria of Aiken and West.[23] sPLS models were programmed in sPLS (https://cran.r-project.org/web/packages/spls/spls.pdf), moderated mediation pathways were programmed in Lavaan (https://cran.r-project.org/web/packages/lavaan/index.html) in the statistical package R 4.1.3.

© 2023 Vaidya N et al. *JAMA Network Open*.

Supplementary Results

eTable 3. Demographics of the cVEDA Cohort

	**cVEDA participants n = 9010**	**Sample used in this study n = 1014**
Age: Median (IQR)	14 (11-18)	15 (11-19)
C1:C2:C3*(%)	32:37:31	26:37:37
Male: n(%)	4303 (47·8)	589 (58·1)
Female: n(%)	4707 (52·2)	425 (41·9)
Education level: Median (IQR)	9 (6-12)	9 (6-13)
SES: Median (IQR)	35 (29-44)	37 (29-47)
BMI: Median (IQR)	18.03 (15.19-21.11)	18.49 (15.66-21.56)

*C1:C2:C3 are age bands for participants within cVEDA divided into following range: 6-11, 12-17, 18-23. SES – Socioeconomic status; BMI – Body Mass Index

eTable 4. Demographics of Participants With and Without Cognitive Data

	**MRI participants n = 1059**	**Sample used in this study n = 1014**
Age: Median (IQR)	15 (11-19)	15 (11-19)
Male: n(%)	608 (57.5)	589 (58·1)
Female: n(%)	451 (42.5)	425 (41·9)
Education level: Median (IQR)	9 (6-13)	9 (6-13)
SES: Median (IQR)	37 (29-47)	37 (29-47)
BMI: Median (IQR)	18.49 (15.66-21.56)	18.49 (15.66-21.56)

SES – Socioeconomic status; BMI – Body Mass Index

eTable 5. Urine Arsenic Levels (μg/L) Among Participants by Site

	**n**	**Arsenic (ug/L): Median (IQR)**
Chandigarh	251	4.6 (2-8.95)
Imphal	30	2.47 (0.26-14.46)
Kolkata (Asansol)	60	3.35 (0.26-9.37)
Rishi Valley	191	8.5 (2.5-16.5)
Bengaluru	482	8.52 (3.25-16.48)
All	1014	6.75 (2.36-14.53)

© 2023 Vaidya N et al. *JAMA Network Open*.

eTable 6. Comparison of Total Urinary Arsenic (μg/L)

Site	N	Age	Urinary arsenic (ug/L) Arithmetic Mean	Geometric Mean (CI-95%)
This Study	255	6-11	9·9	5·1 (4·1-6·9)
	383	12-17	10·4	5·4 (4·6-6·2)
	376	18-23	8·6	4·5 (3·9-5·2)
Canada[24]	514	6-11		5·5 (4·9-6·3)
	506	12-19		5·5 (4·7-6·4)
	362	20-39		5·5 (4·9-6·1)
USA[25]	379	6-11		4·89 (4·29-5·56)
	402	12-19		5·00 (4·52-5·54)
	1794	20+		6·35 (5·88-6·84)
Germany[26]	1734	3-14		4·40 (4·24-4·56)
Belgium[27]	1022	18–80		15·4 (14·2 – 16·8)
United Kingdom[28]	132	Adults	10·48 (Median)	-
China[29]	210	2–12	9·57	-
Bangladesh[30]	312	14-15	205·3	-

© 2023 Vaidya N et al. *JAMA Network Open*.

eFigure 2. Urinary Arsenic Associated With Altered Brain Connectivity

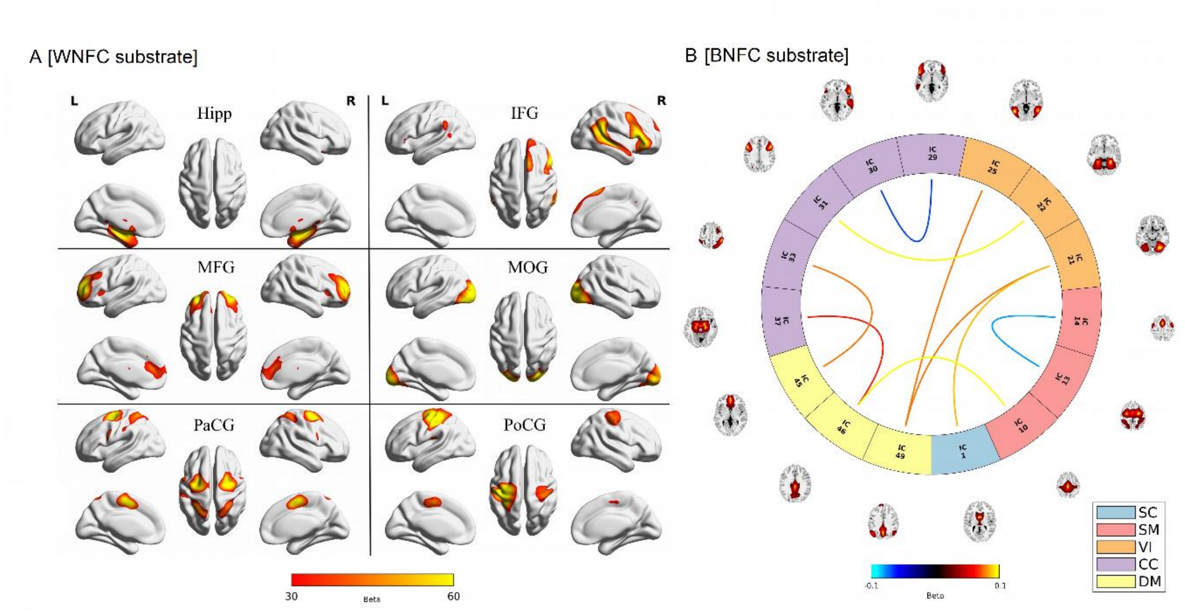

eFigure 2: Urinary arsenic associated with altered brain connectivity.
Panel (a) illustrates the WNFC independent component (IC) maps associated with arsenic; Hipp – Hippocampus, IFG – Inferior frontal gyrus, MFG – Middle frontal gyrus, MOG – Middle Occipital gyrus, PaCG – Paracentral gyrus, PoCG – Postcentral gyrus. Panel (b) is a network graph showing relationship of BNFC and arsenic across domains; SC – Sub cortical, SM – Sensorimotor, VI – visual, CC – Cognitive control, DM – Default mode.

© 2023 Vaidya N et al. *JAMA Network Open*.

eTable 7. Conditional Indirect Effect—Socioeconomic Status

	Brain structure set			WNFC			Cognitive phenotype		
**Effects**	**Coeff**	**SE**	**95% CI**	**Coeff**	**SE**	**95% CI**	**Coeff**	**SE**	**95% CI**
Arsenic	-0·05	0·01	-0·08, -0·03	-0·04	0·01	-0·07, -0·02	-0·008	0·006	-0·02, 0·00
SES	0·04	0·01	0·02, 0·06	-0·008	0·01	-0·02, 0·01	0·01	0·005	0·00, 0·02
Structural substrate							0·05	0·01	0·01, 0·08
WNFC							0·09	0·01	0·05, 0·13
Arsenic * SES	0·03	0·01	0·00, 0·06	0·004	0·01	-0·02, 0·03	0·005	0·007	-0·00, 0·01
**Conditional indirect effect of arsenic on cognitive phenotype by brain structure**									
				**Coeff**		**SE**		**CIs**	
SD below mean				-0·005		0·002		-0·009, -0·001	
Mean				-0·003		0·001		-0·006, -0·001	
SD above mean				-0·001		0·001		-0·004, -0·000	

eTable 8. Conditional Indirect Effect—Body Mass Index

	Brain structure set			WNFC			Cognitive phenotype		
**Effects**	**Coeff**	**SE**	**95% CI**	**Coeff**	**SE**	**95% CI**	**Coeff**	**SE**	**95% CI**
Arsenic	-0·05	0·01	-0·08, -0·03	-0·04	0·01	-0·07, -0·02	-0·009	0·006	-0·02, 0·00
BMI	0·07	0·009	0·05, 0·09	0·003	0·01	-0·01, 0·02	0·007	0·005	-0·00, 0·01
Structural substrate							0·05	0·01	0·01, 0·09
WNFC							0·09	0·01	0·05, 0·12
Arsenic* BMI	0·01	0·01	0·00, 0·04	0·008	0·01	-0·01, 0·03	-0·002	0·005	-0·01, 0·00
**Conditional indirect effect of arsenic on cognitive phenotype by brain structure**									
				**Coeff**		**SE**		**CIs**	
SD below mean				-0·004		0·001		-0·007, -0·001	
Mean				-0·003		0·001		-0·006, -0·001	
SD above mean				-0·002		0·001		-0·004, -0·000	

© 2023 Vaidya N et al. *JAMA Network Open*.

eMethods 3. Sensitivity Analysis

To examine the ecological confounders of arsenic we conducted post hoc analysis using the description of the physical environment of participants as recorded by the environment exposure questionnaire. Bivariate correlation analysis indicated that arsenic was significantly related to proximity of household to agricultural land, engagement in agriculture, exposure to traffic exhaust and insecticides (eTable 9). In our main analysis we controlled for site alone to avoid over adjustment in our model, however, there is a possibility of residual confounding and therefore we ran sensitivity analysis adjusting for participant's physical environment. Even after controlling for these additional confounders, arsenic association with cognition remained significant at p=0.008, r=-0.11.

eTable 9. Correlation Values With Significance Estimating the Relationship of Arsenic With Physical Environment

	**Physical environ-ment**	**Drainage system**	**Landfill of waste**	**Sewage Treatment**	**Traffic exhaust**	**Agricultural land distance**	**Engaged in Agriculture**	**Pesticide exposure**	**Engaged in animal breeding**	**Insecticide exposure**
Arsenic	*r*	-0.03	.068	0.055	.089	.133	.088	.064	0.007	.101
	p	0.27	0.24	0.65	0.03	0.001	0.04	0.33	0.81	0.01

Sensitivity analyses were conducted to identify the most robust associations in mediation models (supplementary table e10, e11). Mediation by the structural component was driven by the right (rostral) anterior cingulate (*b*=-0.001, Cis=-0.02,-0.003), involved in executive control and attention. Mediation by the WNFC component was driven by the medial frontal cortex (*b*=-0.0009, Cis=-0.002,-0.0002), implicated in attentional control and decision making as well as working memory. The remaining structural and functional brain features within the components did not show significant effects individually. Whereas the structural and functional components explained 3.7% and 2.4% of the variance, respectively, rostral anterior cingulate and medial frontal cortex only explained 0.8% and 0.7% of the variance, respectively. These

© 2023 Vaidya N et al. *JAMA Network Open*.

results points towards a mechanistic involvement of two brain regions, whose importance for executive functioning is well established. They also indicate that the overall components captured additional information about the complex relationships between brain function and cognitive outcomes that might have remained undetected when examining the individual variables in isolation.

eTable 10. Mediation Pathways Based on Bootstrapped Variability Around the Product of Nonstandardized Path Coefficient Estimates for Structural Substrate

	**Cognitive phenotype [Estimate (95% CI)]**
**Direct effect**	
Arsenic	- 0·01 (-0·02, -0·00)
**Indirect effect**	
Arsenic via Left parstriangularis	-0·0004 (-0·0002, 0.001)
Arsenic via Left insula	-0.0003 (-0.001, 0.002)
Arsenic via Right inferior temporal	-0.0001 (-0.001, 0.0009)
Arsenic via Right parstriangularis	-0.0009 (-0.0001, 0.002)
Arsenic via Right rostral anterior cingulate	-0.001 (-0.02, -0.003)

eTable 11. Mediation Pathways Based on Bootstrapped Variability Around the Product of Nonstandardized Path Coefficient Estimates for WNFC

	**Cognitive phenotype [Estimate (95% CI)]**
**Direct effect**	
Arsenic	- 0·01 (-0·02, 0·00)
**Indirect effect**	
Arsenic via Left postcentral gyrus	-0.0002 (-0.002, 0.0002)
Arsenic via Paracentral lobule	-0.0007 (-0.002, 0.0002)
Arsenic via Middle occipital cortex	-0.0008 (-0.003, 0.0003)
Arsenic via Right inferior frontal cortex	-0.0005 (-0.002, 0.0001)
Arsenic via Hippocampus	-0.0001 (-0.008, 0.0001)
Arsenic via Middle frontal cortex	-0.0009 (-0.002, -0.0002)

© 2023 Vaidya N et al. *JAMA Network Open*.

eReferences

1. Sharma E, Vaidya N, Iyengar U, et al. Consortium on Vulnerability to Externalizing Disorders and Addictions (cVEDA): A developmental cohort study protocol. *BMC Psychiatry*. 2020;20(1). doi:10.1186/s12888-019-2373-3

2. Zhang Y, Vaidya N, Iyengar U, et al. The Consortium on Vulnerability to Externalizing Disorders and Addictions (c-VEDA): an accelerated longitudinal cohort of children and adolescents in India. *Mol Psychiatry*. 2020;25(8). doi:10.1038/s41380-020-0656-1

3. Sysalova J, Spevackova V. A study of sample mineralization methods for arsenic analysis of blood and urine by hydride generation and graphite furnace atomic absorption spectrometry. *Open Chem*. 2003;1(2). doi:10.2478/BF02479263

4. Flajnik C, Delles F, Marketing VOSI. Evaluation of Deuterium and Zeeman Background Correction with the presence of Spectral Interferences Determinations of Arsenic in an Aluminium Matrix and Selenium in an Iron Matrix by GFAAS. *Agilent Technologies*. Published online 2010:1-8.

5. Zulkifli SN, Yu SM. The food frequency method for dietary assessment. *J Am Diet Assoc*. 1992;92(6):681-685. http://europepmc.org/abstract/MED/1607563

6. Lejuez CW, Read JP, Kahler CW, et al. Evaluation of a behavioral measure of risk taking: The Balloon Analogue Risk Task (BART). *J Exp Psychol Appl*. 2002;8(2). doi:10.1037/1076-898X.8.2.75

7. Corsi PM. *Human Memory and the Medial Temporal Region of the Brain.* ProQuest Information & Learning; 1973.

8. Kessels RPC, van den Berg E, Ruis C, Brands AMA. The Backward Span of the Corsi Block-Tapping Task and Its Association With the WAIS-III Digit Span. *Assessment*. 2008;15(4). doi:10.1177/1073191108315611

9. Croschere J, Dupey L, Hilliard M, Koehn H, Mayra K. The effects of time of day and practice on cognitive abilities: Forward and backward Corsi block test and digit span. *PEBL Technical Report Series*. Published online 2012.

10. Kirby KN, Petry NM, Bickel WK. Heroin addicts have higher discount rates for delayed rewards than non-drug-using controls. *J Exp Psychol Gen*. 1999;128(1):78.

11. Mehta UM, Thirthalli J, Naveen Kumar C, et al. Validation of Social Cognition Rating Tools in Indian Setting (SOCRATIS): A new test-battery to assess social cognition. *Asian J Psychiatr*. 2011;4(3). doi:10.1016/j.ajp.2011.05.014

12. Logan GD, Cowan WB. On the ability to inhibit thought and action: A theory of an act of control. *Psychol Rev*. 1984;91(3). doi:10.1037/0033-295X.91.3.295

13. Piper BJ, Li V, Eiwaz MA, et al. Executive function on the Psychology Experiment Building Language tests. *Behav Res Methods*. 2012;44(1). doi:10.3758/s13428-011-0096-6

14. Berg EA. A Simple Objective Technique for Measuring Flexibility in Thinking. *J Gen Psychol*. 1948;39(1). doi:10.1080/00221309.1948.9918159

15. Fischl B. FreeSurfer. *Neuroimage*. 2012;62(2). doi:10.1016/j.neuroimage.2012.01.021

© 2023 Vaidya N et al. *JAMA Network Open*.

16. Desikan RS, Ségonne F, Fischl B, et al. An automated labeling system for subdividing the human cerebral cortex on MRI scans into gyral based regions of interest. *Neuroimage*. 2006;31(3). doi:10.1016/j.neuroimage.2006.01.021

17. Du Y, Fu Z, Sui J, et al. NeuroMark: An automated and adaptive ICA based pipeline to identify reproducible fMRI markers of brain disorders. *Neuroimage Clin*. 2020;28. doi:10.1016/j.nicl.2020.102375

18. National Family Health Survey [Internet]. Rchiips.org. 2009 [cited 23 November 2020]. Available from: http://rchiips.org/nfhs/about.shtml.

19. Wold S, Eriksson L, Trygg J, Kettaneh N. The PLS method–partial least squares projections to latent structures–and its applications in industrial RDP (research, development, and production). *Unea University*. Published online 2004.

20. Witten DM, Tibshirani R, Hastie T. A penalized matrix decomposition, with applications to sparse principal components and canonical correlation analysis. *Biostatistics*. 2009;10(3). doi:10.1093/biostatistics/kxp008

21. Meinshausen N, Bühlmann P. Stability selection. *J R Stat Soc Series B Stat Methodol*. 2010;72(4). doi:10.1111/j.1467-9868.2010.00740.x

22. Hayes AF. An Index and Test of Linear Moderated Mediation. *Multivariate Behav Res*. 2015;50(1). doi:10.1080/00273171.2014.962683

23. Aiken LS, West SG, Reno RR. *Multiple Regression: Testing and Interpreting Interactions*. sage; 1991.

24. Canada H. Report on human biomonitoring of environmental chemicals in Canada. Results of the Canadian Health Measures Survey Cycle 1 (2007–2009). Published online 2010. Accessed November 23, 2020. https://www.canada.ca/en/health-canada.html

25. CDC. *Fourth National Report on Human Exposure to Environmental Chemicals, Updated Tables.*; 2015. Accessed November 23, 2020. https://www.cdc.gov/exposurereport/

26. UBA. *Levels of Selected Substances in Blood and Urine of Children in Germany. German Environmental Survey for Children 2003/06-GerES (IV) Human Biomonitoring.*; 2008. Accessed November 23, 2020. https://www.umweltbundesamt.de/en/the-uba

27. Hoet P, Jacquerye C, Deumer G, Lison D, Haufroid V. Reference values and upper reference limits for 26 trace elements in the urine of adults living in Belgium. *Clinical Chemistry and Laboratory Medicine (CCLM)*. 2013;51(4):839-849.

28. Morton J, Tan E, Leese E, Cocker J. Determination of 61 elements in urine samples collected from a non-occupationally exposed UK adult population. *Toxicol Lett*. 2014;231(2). doi:10.1016/j.toxlet.2014.08.019

29. Zhang X, Wang B, Cui X, Lin C, Liu X, Ma J. Total arsenic concentrations in Chinese children's urine by different geographic locations, ages, and genders. *Environ Geochem Health*. 2018;40(3). doi:10.1007/s10653-017-9963-0

30. Nahar MstN, Inaoka T, Fujimura M, et al. Arsenic contamination in groundwater and its effects on adolescent intelligence and social competence in Bangladesh with special

reference to daily drinking/cooking water intake. *Environ Health Prev Med*. 2014;19(2). doi:10.1007/s12199-013-0369-z
