# Supplementary material for: Neurocognitive Analysis of Low-level Arsenic Exposure and Executive Function Mediated by Brain Anomalies Among Children, Adolescents, and Young Adults in India
Source: JAMA Netw Open. 2023 May 12;6(5):e2312810. doi: 10.1001/jamanetworkopen.2023.12810 (PMC10182429; doi:10.1001/jamanetworkopen.2023.12810)
Supplement: Supplement 2. — Nonauthor Collaborators [file jamanetwopen-e2312810-s002.pdf]

Supplemental Online Content: Nonauthor Collaborators
*First name, last name, and suffix (if applicable) are required and will appear in PubMed.

*Group Name(s): Consortium on Vulnerability to Externalizing Disorders and Addictions (cVEDA)							
***First Name and Middle Initial(s)**	***Last Name**	***Suffix (eg, Jr, III)**	Academic Degrees	Institution	Location (city, state/province, country)	Role or Contribution, eg, chair, principal investigator	Group (if more than 1 Group listed in the byline) and/or Subgroup (eg, Steering Committee)
Mathew	Varghese			Department of Psychiatry, National Institute of Mental Health and Neurosciences	Bangalore, India	Co-investigator	
Kandavel	Thennarasu			Department of Biostatistics, National Institute of Mental Health and Neurosciences	Bangalore, India	Co-investigator	
Urvakhsh	Metha			Department of Psychiatry, National Institute of Mental Health and Neurosciences	Bangalore, India	Co-investigator	
Satish	Girimaji			Department of Child & Adolescent Psychiatry, National Institute of Mental Health and Neurosciences	Bangalore, India	Co-investigator	
Preeti	Jacob			Department of Child & Adolescent Psychiatry, National Institute of Mental Health and Neurosciences	Bangalore, India	Co-investigator	
Deepak	Jayarajan			Department of Psychiatry, National Institute of Mental Health and Neurosciences	Bangalore, India	Co-investigator	
Keshav	Kumar			Department of Mental Health and Clinical Psychology, National Institute of Mental Health and Neurosciences	Bangalore, India	Co-investigator	
Gitanjali	Narayanan			Department of Clinical Psychology, National Institute of Mental Health and Neurosciences	Bangalore, India	Co-investigator	

Supplemental Online Content: Nonauthor Collaborators
*First name, last name, and suffix (if applicable) are required and will appear in PubMed.

***First Name and Middle Initial(s)**	***Last Name**	***Suffix (eg, Jr, III)**	Academic Degrees	Institution	Location (city, state/province, country)	Role or Contribution, eg, chair, principal investigator	Group (if more than 1 Group listed in the byline) and/or Subgroup (eg, Steering Committee)
Madhu	Khullar			Department of Experimental Medicine, Post Graduate Institute of Medical Education and Research	Chandigarh, India	Co-investigator	
Niranjan	Khandelwal			Department of Radiodiagnosis and Imaging, Post Graduate Institute of Medical Education and Research	Chandigarh, India	Co-investigator	
Abhishek	Ghosh			Department of Psychiatry, Postgraduate Institute of Medical Education & Research	Chandigarh, India	Co-investigator	
Amit				Department of Radiodiagnosis and Imaging, Post Graduate Institute of Medical Education and Research	Chandigarh, India	Recruiter	
Nainesh	Joshi			Department of Experimental Medicine, Post Graduate Institute of Medical Education and Research	Chandigarh, India	Recruiter	
Ningthoujam	Debala Chanu			Regional Institute of Medical Sciences	Imphal, India	Recruiter	
Fujica	M.C.			Regional Institute of Medical Sciences	Imphal, India	Recruiter	
Victoria	Ph.			Regional Institute of Medical Sciences	Imphal, India	Recruiter	
Celina	Phurailatpam			Regional Institute of Medical Sciences	Imphal, India	Recruiter	
Debangana	Bhattacharya			ICMR-Centre on Non-Communicable Diseases	Kolkata, India	Recruiter	
Bidisha	Haque			ICMR-Centre on Non-Communicable Diseases	Kolkata, India	Recruiter	

Supplemental Online Content: Nonauthor Collaborators
*First name, last name, and suffix (if applicable) are required and will appear in PubMed.

***First Name and Middle Initial(s)**	***Last Name**	***Suffix (eg, Jr, III)**	Academic Degrees	Institution	Location (city, state/province, country)	Role or Contribution, eg, chair, principal investigator	Group (if more than 1 Group listed in the byline) and/or Subgroup (eg, Steering Committee)
Alisha	Nagraj			ICMR-Centre on Non-Communicable Diseases	Kolkata, India	Recruiter	
Arpita	Ghosh			ICMR-Centre on Non-Communicable Diseases	Kolkata, India	Recruiter	
Anirban	Basu			ICMR-Centre on Non-Communicable Diseases	Kolkata, India	Lab staff	
Mriganka	Pandit			ICMR-Centre on Non-Communicable Diseases	Kolkata, India	Lab staff	
Subhadip	Das			ICMR-Centre on Non-Communicable Diseases	Kolkata, India	Lab staff	
Pawan	Maurya			ICMR-Centre on Non-Communicable Diseases	Kolkata, India	Lab staff	
Amritha	Gourisankar			Rishi Valley, Rural Health Centre	Rishi Valley, India	Recruiter	
Geetha	Rani T			Rishi Valley, Rural Health Centre	Rishi Valley, India	Recruiter	
Sujatha	B			Rishi Valley, Rural Health Centre	Rishi Valley, India	Recruiter	
Madhavi	Rangaswamy			Department of Psychology, Christ University	Bangalore, India	Co-investigator	
Caroline	Fall			MRC Lifecourse Epidemiology Unit, University of Southamtpon	Southampton, India	Co-investigator	
Kiran	KN			Epidemiology Research Unit, CSI Holdsworth Memorial Hospital	Mysore, India	Recruiter	
Ramya	MC			Epidemiology Research Unit, CSI Holdsworth Memorial Hospital	Mysore, India	Recruiter	

Supplemental Online Content: Nonauthor Collaborators
*First name, last name, and suffix (if applicable) are required and will appear in PubMed.

***First Name and Middle Initial(s)**	***Last Name**	***Suffix (eg, Jr, III)**	Academic Degrees	Institution	Location (city, state/province, country)	Role or Contribution, eg, chair, principal investigator	Group (if more than 1 Group listed in the byline) and/or Subgroup (eg, Steering Committee)
Chaitra	Urs			Epidemiology Research Unit, CSI Holdsworth Memorial Hospital	Mysore, India	Recruiter	
Santhosh	N			Epidemiology Research Unit, CSI Holdsworth Memorial Hospital	Mysore, India	Recruiter	
Somashekhara	R			Epidemiology Research Unit, CSI Holdsworth Memorial Hospital	Mysore, India	Recruiter	
Divyashree	K			Epidemiology Research Unit, CSI Holdsworth Memorial Hospital	Mysore, India	Recruiter	
Arathi	Rao			Division of Nutrition, St. John's Research Institute	Bangalore, India	Recruiter	
Poornima	R			Division of Nutrition, St. John's Research Institute	Bangalore, India	Recruiter	
Saswathika	Tripathy			Centre for Addiction Medicine, National Institute of Mental Health and Neurosciences	Bangalore, India	Recruiter	
Neha	Parashar			Centre for Addiction Medicine, National Institute of Mental Health and Neurosciences	Bangalore, India	Recruiter	
Nayana	K B			Centre for Addiction Medicine, National Institute of Mental Health and Neurosciences	Bangalore, India	Recruiter	
Ashwini	Seshadri			Centre for Addiction Medicine, National Institute of Mental Health and Neurosciences	Bangalore, India	Recruiter	

Supplemental Online Content: Nonauthor Collaborators
*First name, last name, and suffix (if applicable) are required and will appear in PubMed.

***First Name and Middle Initial(s)**	***Last Name**	***Suffix (eg, Jr, III)**	Academic Degrees	Institution	Location (city, state/province, country)	Role or Contribution, eg, chair, principal investigator	Group (if more than 1 Group listed in the byline) and/or Subgroup (eg, Steering Committee)
Sathish	Kumar			Centre for Addiction Medicine, National Institute of Mental Health and Neurosciences	Bangalore, India	Recruiter	
Suneela	Baligar			Molecular Genetics Laboratory, National Institute of Mental Health and Neurosciences	Bangalore, India	Recruiter	
Thamodaran	Arumugam			Department of Neuroimaging and Interventional Radiology	Bangalore, India	Recruiter	
Apoorva	Safai			Department of Neuroimaging and Interventional Radiology	Bangalore, India	Recruiter	
Anthony	Cyril			Centre for Addiction Medicine, National Institute of Mental Health and Neurosciences	Bangalore, India	Recruiter	
Rashmitha				Molecular Genetics Laboratory, National Institute of Mental Health and Neurosciences	Bangalore, India	Recruiter	
Ashika	Roy			Department of Neuroimaging and Interventional Radiology, National Institute of Mental Health and Neurosciences	Bangalore, India	Recruiter	
Dhanalakshmi	D			Centre for Addiction Medicine, National Institute of Mental Health and Neurosciences	Bangalore, India	Support staff	
Shivamma	D			Centre for Addiction Medicine, National Institute of Mental Health and Neurosciences	Bangalore, India	Support staff	
Bhavana	B R			Centre for Addiction Medicine, National Institute of Mental Health and Neurosciences	Bangalore, India	Support staff	
