# Supplementary material for: Neurocognitive Analysis of Low-level Arsenic Exposure and Executive Function Mediated by Brain Anomalies Among Children, Adolescents, and Young Adults in India
Source: JAMA Netw Open. 2023 May 12;6(5):e2312810. doi: 10.1001/jamanetworkopen.2023.12810 (PMC10182429; doi:10.1001/jamanetworkopen.2023.12810)
Supplement: Supplement 3. — Data Sharing Statement [file jamanetwopen-e2312810-s003.pdf]

Data Sharing Statement

Vaidya. Neurocognitive Analysis of Low-Level Arsenic Exposure and Executive Function Mediated by Brain Anomalies Among Children, Adolescents, and Young Adults in India. *JAMA Netw Open*. Published May 12, 2023. doi:10.1001/jamanetworkopen.2023.12810

Data
Data available: Yes
Data types: Deidentified participant data, Data dictionary
How to access data: Details available at https://cveda-project.org/access-to-the-c-veda-dataset/
When available: With publication

Supporting Documents
Document types: None

Additional Information
Who can access the data: Researchers whose proposed use of the data has been approved
Types of analyses: For any research purpose
Mechanisms of data availability: After approval of a proposal with investigator support
